# The relationship between HIV pre-exposure prophylaxis, sexually transmitted infections, and antimicrobial resistance: a qualitative interview study of men who have sex with men

**DOI:** 10.1186/s12889-022-14645-0

**Published:** 2022-11-29

**Authors:** Adam Dale Newman Williams, Fiona Wood, David Gillespie, Zoë Couzens, Kathryn Hughes, Kerenza Hood

**Affiliations:** 1 Centre for Trials Research, School of Medicine, College of Biomedical & Life Sciences, Cardiff University, Wales, UK; 2 Division of Population Medicine, School of Medicine, College of Biomedical & Life Sciences, Cardiff University, Wales, UK; 3 Public Health Wales NHS Trust, Wales, UK

**Keywords:** HIV, Pre-exposure prophylaxis, Sexually transmitted infections, Antimicrobial resistance, Qualitative research, Sexual behaviour, Sexual and gender minorities

## Abstract

**Background:**

HIV pre-exposure prophylaxis (PrEP) is a medication that prevents the acquisition of HIV. It has been targeted towards men who have sex with men (MSM). Since its introduction there have been concerns raised around changes in sexual behaviour such as increased condomless anal intercourse (CAI), leading to an elevation in sexually transmitted infections (STIs). With antimicrobial resistant strains of STIs rising, there are concerns that PrEP may be contributing to this growth. This study aims to understand how MSM conceptualise the relationship between PrEP, STIs and antimicrobial resistance (AMR).

**Methods:**

Twenty semi-structured interviews were conducted online using Zoom. Participants include a mix of PrEP related experiences (never used, currently use, previously used). Reflexive thematic analysis was undertaken by the lead author with 10% of transcripts double coded.

**Results:**

MSM in Wales have positive views and a good knowledge of PrEP and awareness of bacterial STIs. PrEP is perceived by many to lead to a reduction in condom use and increase in STIs but reported condom use behaviours presented to be stable in terms of PrEP initiation. PrEP use is influenced by increased concern for HIV and minimal concern for bacterial STIs. Awareness of AMR STIs was lacking.

**Conclusions:**

There is a belief that PrEP use will lead to an increase in STI rates through reduced condom use, despite reported behaviours often being stable in relation to PrEP initiation, PrEP stigma may be influencing this dichotomy. Concern and awareness for resistant STIs is low, with little association to PrEP.

**Supplementary Information:**

The online version contains supplementary material available at 10.1186/s12889-022-14645-0.

## Introduction/background

HIV pre-exposure prophylaxis (PrEP) is a medication used by HIV negative individuals to prevent HIV. PrEP has been found to have a high efficacy in reducing HIV incidence when taken correctly. PrEP is recommended to either be taken daily or event-driven (2–1-1 method) [1, 2]. In the UK, PrEP has been largely adopted by cisgender men-who-have-sex-with-men (MSM). It has been freely available through the National Health Service (NHS) across the UK since 2020/2021 but Wales was an early adopter, first providing it in 2017. PrEP is also available for personal purchase online. In Wales, PrEP is only recommended by healthcare professionals for individuals at increased risk of HIV and 98% of PrEP users identify as MSM [3]. Eligibility for prescribed PrEP in Wales includes MSM and trans people having sex with men who self-reported on-going condomless anal intercourse (CAI), being HIV negative with a partner who lives with HIV and not virally suppressed or a heterosexual HIV negative person who has condomless sex with people living with HIV of unknown viral suppression [4]. For those using PrEP, regular sexually transmitted infection (STI) testing and renal function monitoring is recommended in Wales [4].

Since PrEP’s introduction there have been concerns that PrEP use would result in increased CAI and subsequently lead to increasing the already rising STI rates [5–8]. This argument of risk compensation related to PrEP is highly debated within the literature, with studies providing both evidence of increases and no change of CAI and STI rates [1, 2, 5–7]. The concern for PrEP potentially increasing STI rates is linked to the growing threat of antimicrobial resistance, particularly for gonorrhoea which is currently under global surveillance due to the increasing identification of antimicrobial resistant strains [9, 10]. This has led to some to fear that increased infection and treatment of gonorrhoea among PrEP users may be a driver of resistance [11].

As the major adopters of PrEP in Wales, an understanding of MSM’s knowledge and concerns is vital to understand sexual decision making and attitudes to STIs within the context of PrEP use and growing antimicrobial resistance (AMR). We explored knowledge and concerns of MSM in relation to PrEP use, contracting an STI, and the increasing cases AMR. Specifically, we aimed to answer the question: how do MSM conceptualise the relationship between PrEP, STIs and AMR.

## Methods

### Design

Qualitative semi-structured interviews were chosen to allow in-depth exploration of participants perception of the relationship between PrEP use, STIs, and AMR. Interviews enabled an open and flexible approach to allow for exploration of participants’ understandings and the ability to adapt throughout data collection. Due to COVID-19 restrictions interviews were conducted virtually.

### Participants

Eligible participants were MSM, aged 18 or over, currently living in Wales. A stratified sampling approach was used, aiming to collect participants with various relationships with PrEP: those currently using PrEP, those who had previously used PrEP and those who had never used PrEP. Recruitment occurred through social media advertisements and targeted through their Grindr profiles. We also used snowball sampling where initial participants helped to identify and recruit other eligible participants. Individuals were excluded if they lacked capacity to give consent, were unable to converse in English or did not have access to an electronic device with which they could participate in an interview.

### Ethics

Ethical approval was granted by Cardiff University School of Medicine Research Ethics Committee. Potential participants showed interest through contacting the study specific email account (indicated on advertisements). These individuals were then sent an information sheet explaining the study. A two-stage consent process was approved by the ethics committee with consent being received via a ‘signed’ electronic consent form and verbal consent recorded at the commencement of the interviews. This two-stage process was adopted at the time in response to research moving to a virtual platform after the COVID-19 pandemic and has since changed.

### Data collection

Video mediated interviews were chosen using the platform Zoom. Remote data collection has the benefit of time efficiency as well as financial and environmental benefits as interviews could be conducted across Wales without the need for travel [12]. Research suggests that video-meditated interviews provide a ‘protective barrier’ that increases comfort when discussing sensitive topic such as sexual behaviour [13]. Participants were thanked for their time with a £20 e-voucher.

We aimed to include between 20 and 25 participants in total, with this sample size informed by the information power model [14] and taking into consideration the specific aims of the research and the strong emphasis on data richness through building trust and rapport with participants. Interviews were conducted following the interview guide developed by the research team, see supplementary material.

### Research team and reflexivity

All interviews were conducted by the lead author (AW). He is a PhD student, and the Chief Investigator of the study with previous experience of conducting and analysing qualitative interviews. AW is a 25-year-old white homosexual cisgender male, who has no lived experience of taking PrEP but experience of the consultation process and regular STI testing. AW was involved in the recruitment for all participants. The similar sexual orientation of the interviewer and interviewees likely improved the insight and quality of information due to the shared experiences. To ensure important perspectives were not missed through unconscious assumptions due to familiarity with the population by AW, the research team was involved throughout to provide wider perspectives and guide the interview schedule and analysis. Ensuring a broad perspective was taken when analysing the data.

### Analysis

All interviews were audio recorded and professionally transcribed verbatim before being imported into NVivo 10 (QSR International) and analysed using reflexive thematic analysis [15, 16]. AW analysed all transcripts with 10% being double coded by co-authors DG and FW to share perspectives and refine coding. Codes were incorporated into broader themes, which were then discussed by the multidisciplinary research team to further aid interpretation.

## Results

Twenty interviews took place between September 2020 and February 2021. All participants were male, aged between 19 and 53 years. The majority were white British (17/20, 85%), gay (17/20, 85%), and single (14/20, 70%). Of those in a relationship, two thirds had an open arrangement (4/6, 66%). Most participants (15/20, 75%) were educated to undergraduate degree level or higher. Participants mostly lived in Southeast Wales (16/20, 80%), mainly in Cardiff (14/20, 70%). Eleven participants reported currently using PrEP (11/20, 55%), with three having previously used PrEP but stopped (3/20, 15%) and six never used PrEP (6/20, 30%). Of those who reported PrEP use, most reported daily use (13/14, 93%), only one participant reported event-driven PrEP use. See Table 1 for full breakdown.

**Table 1:** Characteristics of interviewed participants

		PrEP user		Previous PrEP use		Never used PrEP	
	Variable	[*N* = 11]		[*N* = 3]		[*N* = 6]	
		n	%	n	%	n	%
Sex	Male	11	100.0	3	100.0	6	100.0
Gender	Cis gender	11	100.0	3	100.0	6	100.0
Sexuality	Gay	9	81.8	3	100.0	5	83.3
	Bisexual	0	0.0	0	0.0	1	16.7
	Pansexual	2	18.2	0	0.0	0	0.0
Ethnicity	White British	10	90.9	2	66.6	5	83.3
	White European	1	9.1	0	0.0	0	0.0
	Pakistani	0	0.0	0	0.0	1	16.7
	Mixed	0	0.0	1	33.3	0	0.0
Relationship status	Single	6	54.5	2	66.6	6	100.0
	In a relationship	4	36.4	1	33.3	0	0.0
	Married/Civil Partnership	1	9.1	0	0.0	0	0.0
Relationship type	Open	3	60.0	1	100.0	0	0.0
	Closed	2	40.0	0	0.0	0	0.0
Education level	Postgraduate or equivalent	4	36.4	2	66.6	2	33.3
	Bachelors or equivalent	5	45.5	0	0.0	2	33.3
	A-levels or equivalent	2	18.2	1	33.3	2	33.3
Region of Wales	North	1	9.1	0	0.0	2	33.3
	Southeast	10	90.9	2	66.6	4	66.6
	Southwest	0	0.0	1	33.3	0	0.0

## Knowledge and awareness

### Knowledge of PrEP

Overall, participants had good knowledge about PrEP with accurate and detailed accounts being provided. Their accounts included facts of healthcare approved dosing options (i.e., daily and event driven) and protection only occurring when taken correctly. Some participants cited the effectiveness rates for PrEP, in line with findings produced by the PROUD study and IPERGAY trial [1, 2]. Importantly, participants were clear that PrEP only protected against HIV and no other STIs.

“I would describe PrEP as a medication that can be taken daily or on an incidental basis which can help prevent contracting HIV.” P05 (using PrEP)

Knowledge of PrEP was reported to have been mostly obtained via sexual health clinic nurses, as well as through social interactions amongst other MSM.

### Attitudes/opinions of PrEP

Participants’ opinions of PrEP were universally positive towards the medication. They discussed the numerous ways that PrEP provided freedom to both the individual taking the medication and others within the MSM and wider community. PrEP was valued for its ability to provide “peace of mind” and alleviate the fear of contracting HIV, resulting in less reliance on condoms to protect them from HIV. The wider societal benefits of reducing HIV within the community were also discussed.

“I think I’d always found that sexual health was quite a stressful thing and… the thought of contracting something as serious as HIV, say, when there is an option to, you know, reduce that chance and reduce the chance of it spreading further around the community, is just a good option to take.” P03 (using PrEP)

Both users and non-users agreed that PrEP would improve engagement with sexual health services due to the requirement of regular HIV and STI testing for prescribed users. The health monitoring provided alongside PrEP was perceived as positive benefit for general health maintenance.

“… you have to have contact with a clinician every three months or so. And I think that in terms of testing, in terms of sexual health promotion, in terms of just general wellbeing, and even mental health comes into it, you are being almost monitored.” P07 (never used PrEP)

Social media was conveyed to have had an influence on awareness of PrEP, often by promoting positive information about its use but also propagating PrEP-associated stigmas. Negative representations largely took the form of perceived attacks on the character of those using PrEP with participants reporting others using terms such as “Truvada whore” (P07, never used PrEP) and “PrEP is for tarts that can’t say no.” (P17, using PrEP).

“… particularly in social media, in terms of stigmatisation or to use the phrase, slut shaming, that it can be misrepresented as a free to pass ticket to do what you want. Which is not actually about the drug at all, it’s about the perception of the drug and its users, or misperception.” P04 (using PrEP)

### Knowledge of STIs

Overall, participants had good awareness of STIs with HIV, Chlamydia, Gonorrhoea and Syphilis being commonly discussed. Genital warts, genital herpes and Hepatitis B were less commonly discussed and, where they were mentioned, less detail was provided. Some participants included details of transmissibility and even epidemiological trends of STIs, displaying advanced knowledge.

“Genital warts, probably skin to skin contact. Syphilis is primarily with fluids contact or contact with a… like a chancre that somebody has. The others, blood and fluid born transmission like hepatitis B, hepatitis A. Am I missing anything? Chlamydia, gonorrhoea contact with bodily fluids. I think that's it. Herpes bodily contact.” P06 (Never used PrEP)

“You know, I've seen in my clinic that gonorrhoea is on the… on the up. Syphilis of all things is apparently on the up as well.” P05 (using PrEP)

Many described their knowledge originating from searching for information on the internet in response to personal concerns, receiving a positive diagnosis or experiences of their friends. Many reporting the benefits of being able to so easily search and access information related to sexual health.

“… if I'm concerned about something that is visible or whether I've tested positive for something, then of course I'm going to look it up. Or in instances where my friends have contracted STIs and we’re talking about it, then I'm going to look it up and talk to them to either reassure them or sort of like walk them through the steps and stuff like that.” P20 (using PrEP)

Clinics were described as a source of reliable information which was obtained through leaflets provided by nurses or posters. Most participants described themselves as knowledgeable about STIs.

### AMR awareness

Participants levels of knowledge around AMR varied considerably. Overall, there was a good understanding of antibiotic resistance: knowing it was the microbe (bacteria or virus) that developed resistance to antibiotics. However, there were some reports of the individual or the body becoming resistant to antibiotics. Some participants were aware of public health messaging relating to factors that contribute to antibiotic resistance such as failure to complete a course (although this is heavily debated in scientific literature, [17]) and overuse of antibiotics.

“it’s where antibiotics find that they can resist the antibiotic… sorry, germs, viruses, whatever the organism is, gains resistance by having been subjected to antibiotics of a certain type. But not enough to kill it to a point then which it survives and then recovers and then is able to then multiply again but then has learned that actually, we’re going to learn to defend against that thing that nearly wiped us out. So incorrect use of antibiotics, not taking it for long enough, not taking it at the correct times and dosages.” P01 (never used PrEP)

However, an understanding of AMR did not often correspond with an awareness of AMR in relation to STIs. Many participants voiced surprise when they were made aware of the current global concern for antibiotic resistant gonorrhoea from the interviewer.

Of those who were aware, references to “super gonorrhoea” were often made early in the conversation. The term “super gonorrhoea” has become a popular reference within the UK media and refers to drug-resistant strains of gonorrhoea with high-level resistance to the recommended treatment options (ceftriaxone and azithromycin) as well as many other antibiotics [11]. One participant highlighted an interesting perception that AMR infections, at least for gonorrhoea, are more of a problem within “gay populations.” However, despite the participant citing “outbreaks” in certain UK cities, there is no evidence of these outbreaks occurring within MSM.

“I know that gonorrhoea is one of those illnesses that is becoming too resistant to some forms of treatment. And I also understand that areas with bigger gay populations, particularly London, Manchester, Brighton, tend to have or seem to tend to have bigger outbreaks of resistant gonorrhoea.” P13 (using PrEP)

Participants believed that the awareness of AMR in relation to STIs among the public was poor. Many suggested that improving awareness would be important, with a focus on the consequences of contracting a multi-drug resistant infection.

“I’d probably say that there needs to be less emphasis on actually catching super gonorrhoea and more emphasis on what happens after.” P18 (using PrEP)

Some participants expressed that, even if provided with information relating to AMR STIs, there would be some people (including themselves) who would accept the risk and continue to engage in condomless sex. Participants went on to explain that the problem of antibiotic resistant STIs would need to become a more prominent and direct threat to their own health before they would consider altering their behaviour. No remarks were made relating to concerns about the potential for transmitting resistant bacteria to others, suggesting that either it is not something they are concerned about, or more likely, they are unaware that it is something to they should be concerned about.

“This is where I’m going to be very cynical. I doubt that many things could [stop certain gay men having CAI]. Unless that person has come up against a person or their own experience of that. I’ve… I don’t think that, from the people that I know, and the conversations I’ve had, people would heed many warnings.” P01 (never used PrEP)

## Sexual behaviour

### Sexual health practices

There were mixed views about condom use. Those not using PrEP viewed condoms as essential on a personal and societal level with safety and protection being paramount. Despite their own views of the importance of condoms, it was held that other MSM did not see condoms as important, presenting an ‘irresponsible other’ [18]:

“It should be very important [using condoms], and it should be more widely adopted, that people have sex with protection, irrespective of if they’re on PrEP or not, because of things like chlamydia, gonorrhoea, etcetera. However, do I think that gay men think it [condom use] is important? No.” P01 (never used PrEP)

Of those using PrEP, it was commonly explained that while they would use condoms, they were also comfortable not using condoms, particularly since starting PrEP. Condoms were still viewed as important, but their protection from HIV through PrEP meant the purpose of condoms was now to safeguard from other STIs which were of lower concern.

“… when I wasn’t on PrEP, was using condoms and was significant… and was choosing partners based on their attitudes to condoms. I think PrEP has changed my thoughts on it. … I think I’m willing to use condoms less but know that others may not.” P13 (using PrEP)

Even those participants who did not use condoms out of a personal dislike, still highlighted the benefits within a societal context for safety and reducing transmission.

“It doesn’t mean I like them, but I think they’re important” (P12, using PrEP).

Some acknowledged that their own personal preference for condomless sex was risking the safety and protection of wider society.

“I would say they’re important for somebody who wants… you know, who doesn’t want to contract or… you know, really reduce their risk of catching anything. But for me, I would consider that probably least important because I know that there are treatment options available.” P06 (never used PrEP)

“… Getting tested regularly, especially young LGBT + people” (P10, using PrEP) was essential for many participants and a major element of sexual health maintenance within the culture of MSM. Attitudes towards appropriate frequency was discussed in terms of levels and types of sexual activity. Most participants felt that testing every 3–6 months was adequate, but when at increased risk needed to test more regularly to compensate for potential exposure. For those using PrEP, the 3-month testing may be influenced by PrEP clinic requirements rather than a calculated health decision.

### Risk perception

It was apparent that many participants were aware of their own risk profile. Those reporting low-risk sexual behaviour accurately perceived themselves as at minimal risk to contracting STIs. Those who engaged in higher risk sexual encounters were often aware of the risks they were taking and would adjust other behaviours accordingly, such as increase testing frequency or seeking knowledge of potential infections.

“And then I’d also say just… like just to educate myself just because I did use to be fairly like promiscuous. So, I did look up these things just to know… just to kind of get to grips with what’s out there.” P18 (using PrEP)

### Influence of mental health

Some participants emphasised how their mental health influenced their sexual behaviours. References were made to how sex was used as a coping mechanism to alleviate feelings of depression and loneliness. Consequently, during difficult periods in their lives, individuals reported engaging in higher risk sexual encounters, potentially lowering boundaries, such as relinquishing the use of condoms, just to engage in a physical act of pleasure.

“But the one thing I am very conscious of is that when my mood is bad, I will have more risky sex. And I’m less able to ask for a condom, want a condom, don’t care if I have a condom or not when my mood is particularly bad.” P13 (using PrEP)

## Risk compensation

### PrEP and condoms

Many participants perceived that PrEP use enabled condom-free sex due to PrEP affording protection from HIV. However, it was notable that this was reported as a view of how others would behave, based on friends’ or acquaintances’ experiences, rather than a belief that guided their behaviour.

“… the kind of guys going onto PrEP, …, they do think, I'm taking this one medication invincible, I don’t now need to use condoms now. And they almost forget about everything that was said in that initial meeting when they were given PrEP saying that, you know, you’re only going to be protected against one… one STI, you’re only protected against HIV.” P07 (never used PrEP)

Even those who used PrEP made references to other people believing they do not need to use condoms and despite reporting similar behaviours, they never associated themselves with this group of “others.” Of those using PrEP, there were some who stated outright that they did not like using condoms and would engage in CAI, so PrEP was providing them a protection that they previously did not have.

“I was having condomless sex before PrEP. I think it will now continue to be after PrEP as well.” P05 (using PrEP)

However, most participants who reported a personal reduction in condom use described it to be associated with the onset of PrEP use. On further discussion, it became clear that the reduction occurred mostly with partners with whom they frequently engaged in sexual contact, rather than new partners. In addition, condom use was also affected by partners’ PrEP status; with some participants reporting that they only engaged in CAI with others who use PrEP.

“…with partners I know and trust and are on PrEP then I don’t necessarily have to use condoms, but for new partners who I don’t trust and haven’t got, you know, information about their sexual status, then I do use condoms.” P05(using PrEP)

For participants who use PrEP, the function of condoms changed with the initiation of PrEP such that condoms were primarily seen as protection from STIs rather than STIs and HIV. The consequence of this is that, in this context, condoms were seen as less important.

“… they use condoms because the biggest worry tends to be HIV. And so, when you take out the HIV by being on PrEP, you remove… you remove the thought of HIV, and then people are more likely to have condomless sex…” P10 (using PrEP)

### Impact on STIs and AMR

All participants commented that they thought there was a relationship between PrEP and STIs, suggesting PrEP increases STIs, particularly chlamydia and gonorrhoea. The increase was perceived to be due to the reduction in condom use, reported both among those using PrEP and not, which would lead to a spread in other infections.

“… if I was to try and draw any correlation between it in my head it’s that, you know, before PrEP, I never had any STIs. After having a couple of years of PrEP, …, STIs seem to come from left, right and centre. So, the correlation I would draw in my head is that PrEP has meant that chlamydia and gonorrhoea have spread a lot faster.” P03 (using PrEP)

As PrEP provided a protection from HIV, the occasional STI seemed an acceptable “trade-off” (P13, using PrEP), with chlamydia and gonorrhoea even being viewed as “occupational hazards” (P13, using PrEP). Some did identify that STIs were rising before PrEP due to reducing condom use among MSM and PrEP may have exacerbated an already growing issue.

“I wouldn’t be surprised if rates of other STIs go up. Because of the people who think that they can have a free pass. However, I wouldn’t be surprised if they’re already rather high because of the naivety of some gay men and the issues regarding the relationship with sex in the gay community.” P01 (never used PrEP)

Some participants expressed hope that the regular testing required by PrEP users would detect and counteract any potential increase in STIs. With identification and treatment occurring sooner, spread among the community would be reduced.

“I just hope that regular testing picks it up, but I would not be surprised if there was an increase in particularly the bacterial… the very easy to transmit bacterial infections such as chlamydia and gonorrhoea. But then again, they were always very easy to spread through oral sex, through rimming, through just touching someone’s penis, you can pick it up. I don’t know about syphilis. I mean, I wouldn’t be surprised if that also increased. But I would imagine it would… it could only go up with PrEP usage. But that feels like… if that is manageable that feels like a small price to pay for the possibility of eradicating HIV, which is by far the most deadly, the most expensive for the NHS to treat, the most impactful on life and on a healthy life.” P13 (using PrEP)

Participants had less certainty around how PrEP may influence AMR. Few associated the rise in STIs with potentially driving antibiotic resistance among infections such as gonorrhoea and chlamydia.

## Discussion

Our qualitative study indicates that MSM may have a good understanding of PrEP and why it is of use in key populations such as MSM. For our participants, PrEP was valued for its impact on reducing HIV as well as reducing anxiety over HIV acquisition; a finding found in several previous studies [19–21]. In our study, MSM also had a reasonable knowledge of many STIs, with HIV, gonorrhoea and chlamydia being most familiar to participants. In addition, many participants had a basic understanding of AMR, although there was less clarity of how AMR related to STIs.

Condoms were viewed to be important by many of our participants, although some viewed their importance to be decreasing since the advent of PrEP. Participants considered that PrEP use would lead to a decrease in condom use among PrEP users. While many PrEP users did discuss their reduction in condom use, it was mostly with regular partners. Consequently, PrEP did not replace condom use, but allowed for flexibility of condom use in certain circumstances. Previous research found similar findings of self-reported reduction in condom use after PrEP initiation [6, 7]. However, as sustained CAI is a required risk factor to receive PrEP from the NHS, it is difficult to know if a reduction of condom use has occurred in a group who, by definition, are inconsistent condom users. For those acknowledging an increase in CAI, the increased testing for STIs was viewed to counterbalance the increased risk. Risk of HIV infection was the paramount issue, and for some resulted in reduced concern towards protection from other infections once PrEP was initiated. While some participants were aware of AMR STIs, this was not viewed as a current and immediate risk but felt that resistant STIs would need to be more prevalent and untreatable before they would consider changing their behaviour. The belief that AMR STIs were not a personal risk was also identified in qualitative work conducted in Bristol among PrEP users [19]. This suggests that the problem of STI AMR would need to become a personal, direct, and serious threat before condoms are used alongside PrEP.

Our study illustrates how PrEP has been adopted as one of many HIV risk-reduction approaches, providing protection for those who are likely to be at risk of acquiring HIV. As in other studies [8, 20, 21], PrEP was also valued for significantly reducing HIV fear and anxiety, improving emotional wellbeing, intimacy, and sexual liberation. However, the issues surrounding PrEP stigma are ever present and pose a threat to initiation and adherence [22]. Tackling this stigma may be key to ensuring vulnerable groups are able to benefit from the provision of PrEP.

Our study shows that the relationship between PrEP, CAI and STIs is complex and not as straightforward as hypotheses around risk compensation would suggest [23]. Data suggests condom use behaviours are stable as those who engaged in CAI before initiating PrEP continue to do so after initiation, while condom users continue their use and have less desire to access PrEP. Even among those PrEP users reporting CAI, it was not with all partners and condom use was often regulated by familiarity and the partners PrEP status. The difference between perceived CAI and reality reported may be due to PrEP associated stigma altering PrEP users’ perceptions of their condomless episode frequencies. However, social desirability bias may also be influencing the reporting of CAI. With people putting conditions on their CAI when discussed to improve their perceived image with the interviewer.

We noted that participants perceived bacterial STIs as easily treatable, and this seems to result in a low level of concern for their transmission. With the increases in AMR, it is essential that public health measures are taken to reduce behaviours that spread bacterial STIs such as gonorrhoea [23]. The low awareness of AMR in relation to STIs suggests the need for interventions related to improved AMR education which confronts the potential future realities of treatment-resistant infections.

### Strengths and limitations

This work was conducted during the COVID-19 pandemic with interviews being transitioned to a virtual platform. Despite this adaption, we have no evidence that remote interviews resulted in poor rapport with our participants. Indeed, it may have been beneficial for participants to be able to discuss their sexual behaviour from the privacy of their homes and this may have resulted in a more honest and open exchange of views. Remote interviews also enabled ease of data collection across Wales. However, most participants lived in Cardiff, and this may have biased the sample towards MSM from an urban setting. Although, it is well documented that LGBTQ + populations tend to congregate within big cities [24]. So, participants mostly living in Wales’ capital city may not be overly biased. It is worth noting that data collection took place during the COVID pandemic and social restrictions, therefore at a period of heightened public awareness of health behaviour and health messaging from government and the scientific/ health professional community. This might have primed our participants to reflect on their health behaviour more than they may have previously done.

We used three methods of sampling to create a varied sample. Our recruitment method via social media advertising and Grindr will have limited the sample to only those who typically use the Grindr application. However, we attempted to address this potential bias through recruiting by snowball sampling. We do, however, acknowledge that this method also can be limited due to similar opinions and behaviours being shared amongst friendship groups.

Interviews about sexual health and behaviours are sensitive and require trust and rapport between participant and interviewer. AW (interviewer and primary analyst) being a gay male helped with rapport building and gave participants a level of comfort due to the interviewer’s familiarity with the cultural norms of sexual behaviour among the population. However, being immersed within the culture is also a limitation and may have resulted in certain topics not being adequately explored due to presumed shared knowledge between interviewer and participant. Double coding was completed by heterosexual colleagues which we hope has gone some way to encourage the discussion of different interpretations and understandings.

Our sample included a range of MSM participants: (people using PrEP, previous PrEP users and those never having used PrEP) providing a mixture of perspectives and strengthening the conclusions drawn from the analysis. However, the sample was dominated by well-educated individuals, and it is the level of knowledge is unlikely to reflect the understanding and concerns of MSM across Wales. The sample also lacked ethnic diversity, which limits the scope of findings.

## Conclusion

MSM in Wales have positive views and a good knowledge of PrEP. PrEP is perceived by many to lead to a reduction in condom use, but this was found to be only in specific scenarios. Any reduction was influenced by the concern placed on HIV being eased and minimal concern for bacterial STIs. Clinicians who provide PrEP to at-risk populations should be mindful to explain that other STIs are also of importance and are becoming harder to treat with the intention of increasing awareness of bacterial STIs.

Although MSM are primarily concerned with protecting themselves from HIV, there is a need to improve the public health messaging around other STIs and AMR. Although this paper has focussed on perceptions of MSM, it is likely that poor understanding of AMR STIs is common across other at-risk groups as in general society. The current focus on health behaviours highlighted by the COVID-19 pandemic may be an opportunity to improve these messages at a population level.

## Supplementary Information

**Additional file 1.**

## Data Availability

The datasets generated and/or analysed during the current study are not publicly available due to limitations of ethical approval involving the patient data and anonymity but are available from the corresponding author on reasonable request.
