# Supplementary material for: The relationship between HIV pre-exposure prophylaxis, sexually transmitted infections, and antimicrobial resistance: a qualitative interview study of men who have sex with men
Source: BMC Public Health. 2022 Nov 29;22:2222. doi: 10.1186/s12889-022-14645-0 (PMC9708133; doi:10.1186/s12889-022-14645-0)
Supplement: Supplementary file 1 — Additional file 1. [file 12889_2022_14645_MOESM1_ESM.docx]

**Supplemental materials:**

UPrEP Qualitative Interviews: Interview Schedule

Hello, my name is XX, I am the main researcher for the XX study. Thank you for agreeing to speak to me today. I am looking to gain an understanding of your awareness and any concerns around PrEP, STIs and antimicrobial resistance. Please remember that everything you say to me will be kept strictly confidential. If you find any of the topics difficult, please let me know. If you wish to decline an answer you may do so. Any questions before we start? Please confirm your consent to the study and to be audio recorded by saying your name and the date.

This interview is being conducted by XXX with (participant ID) on (date) commencing (time).

***Topic 1:***

*First, I would like to discuss PrEP.*

• How would you describe what PrEP is to someone?

• How did you first hear about PrEP?

• What encouraged your decision to start taking/not take PrEP?

- For those who have stopped PrEP – What were your reasons for stopping PrEP? Will you start again?
- Those using PrEP - Have you ever stopped taking prep? Reasons?

• What benefits do you believe there are to PrEP for you and others?

• Can you think of any issues/problems with PrEP?

*There are suggestions that PrEP use could reduce condom use.*

• What do you think of their suggestion?

- Query their response with why?
- Do you think your condom use has changed since starting PrEP?

• In your opinion, do you think condoms are important?

• From your experience, do you think gay and bisexual men are concerned with protection from sexually transmitted infections?

• Would you say there are differing levels of concern?

***Topic 2:***

*Now if we could discuss sexually transmitted infections.*

• How well do you think your knowledge is?

• Can you tell me a bit about some of the STIs you know about and your knowledge of how they can be passed on?

• Where do you get your knowledge from? Proactively looking?

• When entering, or considering, a sexual encounter with someone, what safety measures do you typically think about?

• Are you concerned about catching an STI?

- - What do you think informs your level of concern?

• What impact, if any, do you think PrEP may have on STIs?

• What do you think the rate of STIs in gay and bisexual men is?

• Can you think of any ways to reduce STI rates among gay and bisexual men?

- - Easier/quicker access to testing?
  - More information about STIs?
  - More information about benefits of condoms?
  - More information about rates of STI infections in the community?

***Topic 3:***

*Moving to the next topic.*

• Are you aware of the term antimicrobial or antibiotic resistance?

- Could you explain your understanding of it?
- [If yes] could you explain where you have heard about this or what in relation to?
- [If no] provide brief explanation

• Are you concerned about AR STIs?

- Do you think others are concerned?

• Do you think there is much awareness of antibiotic resistant?

- What about for STIs particularly?

• Do you think being made aware of antibiotic resistant STIs and its consequences would alter sexual practices among gay and bisexual men?

• What do you think would be the best way to inform gay and bisexual men about antibiotic resistant sexual infections?

***Topic 4:***

*Lastly, do you think Covid-19 will have any long-lasting impacts on sexual behaviour or concerns around STIs?*

*Is there anything else you think I should know or want to add?*

*That concludes out interview, thank you for your time.*
